# Supplementary material for: MetaRibo-Seq measures translation in microbiomes
Source: Nat Commun. 2020 Jun 29;11:3268. doi: 10.1038/s41467-020-17081-z (PMC7324362; doi:10.1038/s41467-020-17081-z)
Supplement: Supplementary file 10 — Supplementary Data 7 [file 41467_2020_17081_MOESM10_ESM.zip › File2/Confidence_High_Taxonomy/357470_out.krona.html]

Javascript must be enabled to view this page.

members
magnitude
magnitudeUnassigned
count
unassigned
taxon
rank

357470\_out

3

superkingdom
2
3

phylum
3
976

class
200643
3

order
171549
3

family
171552
3

genus
3
838

2292054
3

SRS012849\_contig\_number\_20258SRS023526\_contig\_number\_12685SRS077849\_contig\_number\_27982
species
